# Uterine tumours resembling ovarian sex cord tumours: a case report

**DOI:** 10.1186/1757-1626-2-55

**Published:** 2009-01-14

**Authors:** Omar Aziz, John Giles, Simon Knowles

**Affiliations:** 1 Gynaecology Department, Yeovil District Hospital, Higher Kingston, Yeovil, Somerset BA21 4AT, UK

## Abstract

**Background:**

Endometrial stromal tumours with sex-cord-like elements were first described by Clement and Scully in 1976. The recent World Health Organisation classification 2003 recognises low grade stromal sarcoma and undifferentiated endometrial sarcoma.

**Case presentation:**

A 62-year-old caucasian woman presented with recurrent postmenopausal bleeding. She underwent a diagnostic hysteroscopy which showed a large polyp on the posterior uterine wall. Polypectomy was performed and the histological report of the polyp showed a complex adenomatous lesion of uncertain malignant potential. She underwent a total abdominal hysterectomy and bilateral oophorectomy. The patient is asymptomatic at the time being, and is having annual check ups.

**Conclusion:**

Total abdominal hysterectomy and bilateral ovarian oophorectomy is the best approach at this stage.

These tumours should be considered as having an uncertain but low likelihood of recurrence. It is acknowledged that no extra uterine spread or distant metastases have been reported thus far.

## Background

Endometrial stromal tumours with sex-cord-like elements are relatively rare (account for 0.25% of all uterine malignancies). These tumours were first described by Clement and Scully in 1976 who presented 4 cases of such tumours and classified them into groups I and II. The groups are defined by the amount of sex cordlike elements.[1] The recent World Health Organisation classification 2003 recognises low grade stromal sarcoma and undifferentiated endometrial sarcoma. Low grade sarcomas may exhibit other forms of differentiation, including smooth muscles and sex cord differentiation. In the latter form, the tumour contains epithelial-like or sex cord like elements, often with epithelioid appearance, arranged in nests, cords, trabeculae, solid, or tubular structures. If this element predominates, the tumour is considered to be a uterine tumour resembling ovarian sex cord tumour(UTROSCTs), and may cause diagnostic difficulties.

## Case report

We report this rare case of carcinoma of the endometrium in a 62-year-old woman who presented with recurrent postmenopausal bleeding. Her medical condition was unremarkable. She underwent a diagnostic hysteroscopy which showed a large polyp on the posterior uterine wall. Polypectomy was performed and the histological report of the polyp showed a complex adenomatous lesion of uncertain malignant potential, therefore she had a standard pelvic MRI staging scan which revealed no evidence of pelvic lymphadenopathy, cystic changes in the cervix and a lesion on the left side of the fundus which appeared to be associated with some thinning of the endometrium which could represent invasion, however, it was not full thickness and the MRI excluded extra-uterine spread.

The patient underwent a total abdominal hysterectomy and bilateral oophorectomy. The pelvis looked clear; both ovaries looked healthy with no lymphadenopathy, and peritoneal washings were taken and sent for cytology.

**Figure 1:**
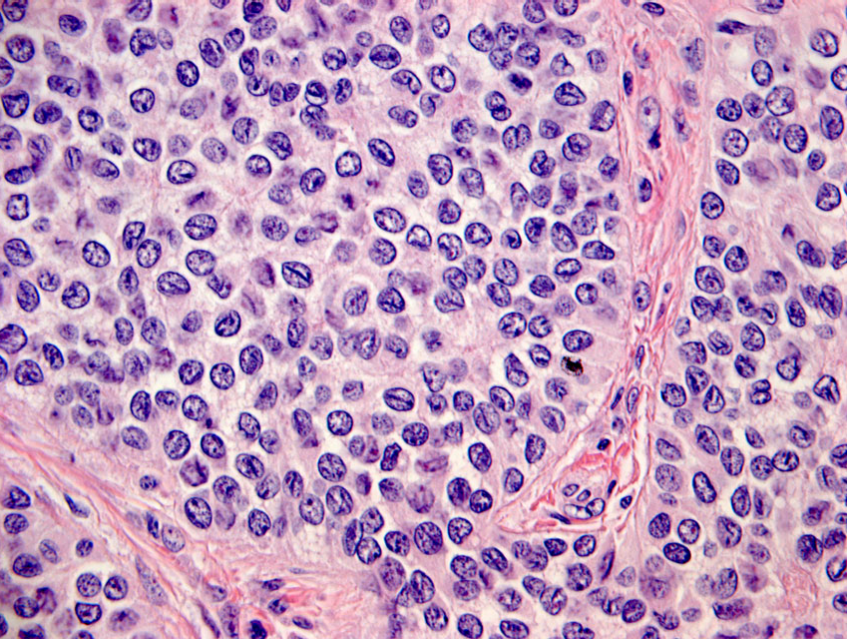
**Microscopic image of the polyp found in the endometrial cavity**.

Macroscopically the uterus showed a large soft peach coloured polyp in the endometrial cavity 20 × 9 × 20 mm and a small yellow deposit in the myometrium 3 mm in size 2 mm adjacent to the polyp. Microscopically, the polyp was composed of cord, nests, trabecular and occasional glandular structure that were formed by small tumour cells that resembled the granulosa cells of ovarian granulosa cell tumours (see Figure 1). Occasional luteinized like cells with abundant foamy cytoplasm were seen only focally, thought to reflect leydig cell differentiation. The tumour immunostained with antibodies that recognize sex cord elements(inhibin, calretinin and CD99), epithelial differentiation (AE1/3 and MNF1165, but not EMA) and myoid (a mesenchymal marker) and WT1, but not CD10, a marker of endometrial stromal cells. All tumour cells expressed oestrogen and progesterone strongly.

At the present, the patient is asymptomatic and is undergoing a yearly check up with pelvic examination and ultrasound scanning which have been normal so far.

## Discussion

There are 2 uterine neoplasms that contain endometrial stroma and are variants of the usual endometrial stromal tumour. One is termed as uterine neoplasm resembling an ovarian sex cord tumour(UTROSCTs), and the other one, a combined smooth muscle stromal tumour[2]

Most papers in the literature consider UTROSCTs to be rare variants of endometrial stromal tumours. Since they were first described 30 years ago, the rarity and limited follow up does not provide robust evidence upon which to base a definite management strategy. In the most recent WHO publication, these tumours have been separated from the main group of endometrial stromal and related tumours and placed in the category of "miscellaneous tumours", however, there is no guidance as to predictive or prognostic factors for the behaviour of these tumours in the current WHO fascicle, the management of these tumours is therefore debatable. One case report states that there is a potential for recurrence or even a metastasis especially if the tumours are invasive [3]

## Conclusion

Due to the lack of evidence about the best management for this type of tumour, total abdominal hysterectomy and bilateral ovarian oophorectomy is the best approach at this stage.

The long-term clinical behavior of UTROSCT remains to be established. Although favorable histological features including well-circumscribed borders and an absence of vascular invasion are usually present, these tumors may on occasion show infiltrative borders and focal vascular invasion, albeit not to the extent that is characteristic of endometrial stromal sarcomas[3]. In the same study four of the cases have had favourable outcomes, but the duration of follow up was limited. The authors therefore advocate that these tumours be considered as having an uncertain but low likelihood of recurrence. It is acknowledged that no extra uterine spread or distant metastases have been reported thus far.

## Abbreviations

UTROSCTs: Uterine Tumours Resembling Sex Cord Tumours; CD: Cluster designation (cluster of differentiation).

## Consent

A written consent has been obtained from the patient.

## Competing interests

The authors declare that they have no competing interests.

## Authors' contributions

OA wrote the original paper. JG treated the patient, revised and edited the manuscript. SK analysed pathology slides, revised and edited the manuscript.
